# Ramipril and Losartan Exert a Similar Long-Term Effect upon Markers of Heart Failure, Endogenous Fibrinolysis, and Platelet Aggregation in Survivors of ST-Elevation Myocardial Infarction: A Single Centre Randomized Trial

**DOI:** 10.1155/2016/9040457

**Published:** 2016-03-15

**Authors:** Martin Marinšek, Andreja Sinkovič

**Affiliations:** Department of Medical Intensive Care, University Clinical Centre Maribor, Ljubljanska 5, SI-2000 Maribor, Slovenia

## Abstract

*Introduction*. Blocking the renin-angiotensin-aldosterone system in ST-elevation myocardial infarction (STEMI) patients prevents heart failure and recurrent thrombosis. Our aim was to compare the effects of ramipril and losartan upon the markers of heart failure, endogenous fibrinolysis, and platelet aggregation in STEMI patients over the long term.* Methods*. After primary percutaneous coronary intervention (PPCI), 28 STEMI patients were randomly assigned ramipril and 27 losartan, receiving therapy for six months with dual antiplatelet therapy (DAPT). We measured N-terminal proBNP (NT-proBNP), ejection fraction (EF), plasminogen-activator-inhibitor type 1 (PAI-1), and platelet aggregation by closure times (CT) at the baseline and after six months.* Results*. Baseline NT-proBNP ≥ 200 pmol/mL was observed in 48.1% of the patients, EF < 55% in 49.1%, and PAI-1 ≥ 3.5 U/mL in 32.7%. Six-month treatment with ramipril or losartan resulted in a similar effect upon PAI-1, NT-proBNP, EF, and CT levels in survivors of STEMI, but in comparison to control group, receiving DAPT alone, ramipril or losartan treatment with DAPT significantly increased mean CT (226.7 ± 80.3 sec versus 158.1 ± 80.3 sec, *p* < 0.05).* Conclusions*. Ramipril and losartan exert a similar effect upon markers of heart failure and endogenous fibrinolysis, and, with DAPT, a more efficient antiplatelet effect in long term than DAPT alone.

## 1. Introduction

Blocking the renin-angiotensin-aldosterone system in ST-elevation myocardial infarction (STEMI) patients prevents heart failure and recurrent thrombosis in particular by the use of angiotensin-converting-enzyme (ACE) inhibitors if there are no contraindications to their use [1, 2]. Early after STEMI they significantly improve outcomes, but according to guidelines their long-term use does not seem mandatory in asymptomatic STEMI patients without left ventricular systolic dysfunction or diabetes [1, 2]. An alternative to ACE inhibitors are angiotensin receptor blockers (ARBs) as demonstrated by the OPTIMAAL trial (Optimal Trial in Myocardial Infarction with the Angiotensin II Antagonist Losartan) [3].

Previous studies demonstrated similar short-term effects of losartan and ramipril in STEMI patients on markers of heart failure such as NT-proBNP and ejection fraction (EF), as well as on markers of endogenous fibrinolysis such as PAI-1 [4].

Regarding the effect on PAI-1 in the long term, studies indicated that ramipril seemed more efficient [3].

In hypertensive patients, ACE inhibitors prevent platelet aggregation, which is an important mechanism for recurrent coronary thrombosis [5–8]. Some ARBs, including losartan, exert an antiplatelet effect such as inhibition of platelet thromboxane A2-induced platelet aggregation as it was demonstrated in hypertensive patients [9]. In addition, losartan specifically prevents platelet adhesion by p-selectin blockade [9, 10].

In STEMI patients, in particular after primary percutaneous coronary intervention (PPCI), high residual platelet reactivity is associated with increased risk of recurrent coronary thrombosis despite of dual antiplatelet therapy [11, 12]. Residual platelet reactivity can be monitored by several methods, including measuring closure times (CT) and being a simple, rapid assessment of high shear-dependent platelet function in whole blood, including platelet adhesion, activation, and aggregate formation [13–15].

Our goal was to evaluate whether six-month treatment by ramipril and losartan exerted any effect on PAI-1, ejection fraction (EF) of the left ventricle, and NT-proBNP and any antiplatelet effect, as measured by CT for the collagen/epinephrine (CEPI) in survivors of STEMI who were treated by PPCI and dual antiplatelet therapy (DAPT).

## 2. Methods

The study was approved by the National Ethical Committee of the Republic Slovenia (69/10/98). Written informed consent was obtained from all included patients. The study protocol conformed to the ethical guidelines of the Declaration of Helsinki. The study was registered by Ema Europe (EudraCT number 2016-000243-14).

### 2.1. Patients Studied

We included patients with their first acute STEMI, admitted to the Department of Medical Intensive Care after PPCI was performed at the catheterization laboratory. Exclusion criteria were shock, severe pulmonary edema, hypotension, bronchospasm, severe infection with sepsis, acute renal and respiratory failure, prior treatment with ACE inhibitors or ARBs, and refusal [2].

After receiving written informed consent we randomized the included patients in a double-blind random fashion in to either losartan or ramipril groups—30 patients to ramipril, titrated to 10 mg daily, and 32 patients to losartan, titrated to 100 mg daily, according to blood pressure measurements. Seven patients discontinued therapy. Finally, we studied 28 patients who were randomly assigned ramipril and 27 who were assigned losartan, receiving therapy for six months.

In addition, the antiplatelet activity of the studied groups was compared to a small control group of 9 STEMI patients, treated only by DAPT without blocking the renin-angiotensin-aldosterone system. Dual antiplatelet therapy consisted of acetylsalicylic acid (ASA) and clopidogrel or ticagrelor or prasugrel.

### 2.2. Study Design

Our hypothesis was that no differences existed between the ramipril and losartan group of STEMI patients. To confirm the null hypothesis that no large effect size existed between the two studied groups, a sample size of more than 25 cases per group was needed (power = 0.8, alpha = 0.05).

In this prospective, randomized, double-blind monocenter study conducted at the Department of Medical Intensive Care of the University Clinical Centre Maribor in Slovenia, the studied STEMI patients were included within the first 24 hours of an in-hospital stay. Before randomization the patients were treated by PPCI and received all the treatments according to current ESC guidelines: ASA, an additional oral antiplatelet agent (either clopidogrel or ticagrelor or prasugrel), statin, and a beta blocker if indicated [1, 2, 12].

STEMI was additionally confirmed by the rise and fall of troponin I [1, 2, 16].

At the start of the study, pretreatment data were recorded, including age, gender, body mass index (BMI), prior arterial hypertension, diabetes, anterior location of acute STEMI, heart failure of Killip class ≥ II before randomization, treatment with PPCI, and treatments by oral antiplatelet agents (ASA with clopidogrel or prasugrel or ticagrelor).

At the start of the study, prior to randomization, and six months later, a physical examination and echocardiography were conducted, and blood samples were drawn.

Echocardiography was performed on an Phillips HDI 3000 ultrasound machine. We measured the ejection fraction (EF) by a modified biplane Simpson's method. The normal level for EF was 55% [17–19].

During the follow-ups over the next 6 months all the complications were recorded, in particular heart failure, which was defined as classes II–IV according to the Killip-Kimball classification [1, 2, 12]. Killip class II was characterized by protodiastolic gallop and/or tachycardia and pulmonary rales in the lungs were registered. In Killip class III signs of pulmonary edema were present, and in Killip class IV there were signs of cardiogenic shock [2, 19].

In case of pulmonary edema or cardiogenic shock the patients were excluded from the study and treated according to guidelines by the treating physician [2].

### 2.3. Blood Samples and Laboratory Methods

Blood samples to measure PAI-1 activity were drawn before and 6 months after randomization between 8:00 and 10:00 a.m. Blood samples were centrifuged and plasma was frozen and stored at −70°C. PAI-1 activity was measured by the chromogenic method (normal levels 0.3–3.5 U/mL, Berichrom PAI by Dade Behring, Marburg, Germany) [20, 21].

Blood samples to measure NT-proBNP were drawn just prior to and 6 months after randomization between 8:00 and 10:00 a.m. Plasma NT-proBNP levels were measured by the electrochemiluminescence immunoassay on an Elecsys 2010 analyzer (Roche Diagnostics, normal levels up to 20 pmol/L) [21, 22].

Blood samples to measure troponin I were drawn on hospital admission and once per day over the first few days after PPCI. Troponin I was measured by the immunochemical method (Siemens Healthcare Diagnostics Inc., Newark, USA, normal levels up to 0.045 *μ*g/L) [2, 21]. Total serum cholesterol, HDL-cholesterol, and triglycerides were measured by the colorimetric method (Ektachem 250 Analyzer, Eastman Kodak Company, Rochester, USA). LDL-cholesterol level was measured by homogeneous assays [23]. The lipid profile was measured upon admission and after 6 months of treatment.

Platelet count measured by an automatic counter, the Sysmex XE-2100, Kobe, Japan; normal levels were 140–340 × 10^9^/L upon admission, after 8 weeks, and after 6 months [24].

Residual platelet reactivity was measured by closure times (CT), using a platelet function analyzer device (PFA-100®). PFA-100 CT enabled the simple, rapid assessment of high shear-dependent platelet function. Small amounts of citrated blood were needed (0.8 mL/cartridge; maximal CT results: 300 s) [13–15]. Blood samples were aspirated at high shear rates (5000–6000 s) through a capillary in the instrument cartridge to meet a membrane coated with collagen/epinephrine (CEPI) [13–15]. The membrane triggered platelet adhesion, activation, and aggregate formation, leading to occlusion of the membrane and cessation of blood flow [13–15]. Normal CT levels for CEPI were 82–150 seconds, but CT values > 300 seconds were nonclosure [13–15, 25].

### 2.4. Statistical Analysis

Statistical analyses were performed using the SPSS® statistical package, version 19 (SPSS Inc., Chicago, IL, USA) for Windows®. Data were expressed as mean ± standard deviations or percentages. Differences between the groups were tested by the two-sided Student's *t*-test for mean ± standard deviations and by the chi-square test for percentages. A *p* value < 0.05 was considered statistically significant.

## 3. Results

Baseline clinical and laboratory data of all included STEMI patients, as well as ramipril and losartan treated STEMI patients, are summarized in Table 1.

Between patients treated with ramipril and losartan there were nonsignificant differences in baseline clinical and laboratory data as illustrated in Table 1.

**Figure 1:**
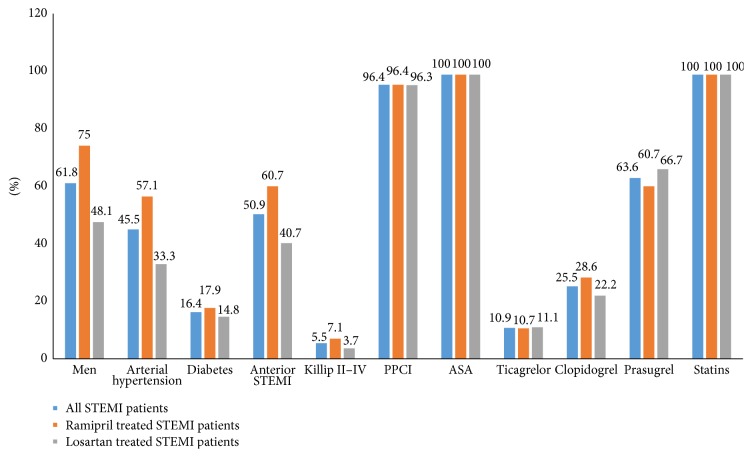
Baseline characteristics of all STEMI patients and comparison between ramipril and losartan treated STEMI patients. STEMI: ST-elevation myocardial infarction; PPCI: primary percutaneous coronary intervention; ASA: acetylsalicylic acid.

Comorbidities, the use of PPCI, and the use of antiplatelet agents are displayed in Figure 1. There were nonsignificant differences between the studied groups as shown in Figure 1.

Table 2 shows mean levels of NT-proBNP, EF, and PAI-1 at baseline and six months after randomization. Between ramipril and losartan there were only nonsignificant differences. Within ramipril and losartan group NT-proBNP decreased significantly within 6 months in comparison to the baseline, but mean PAI-1 and EF levels changed nonsignificantly as shown in Table 2.

**Figure 2:**
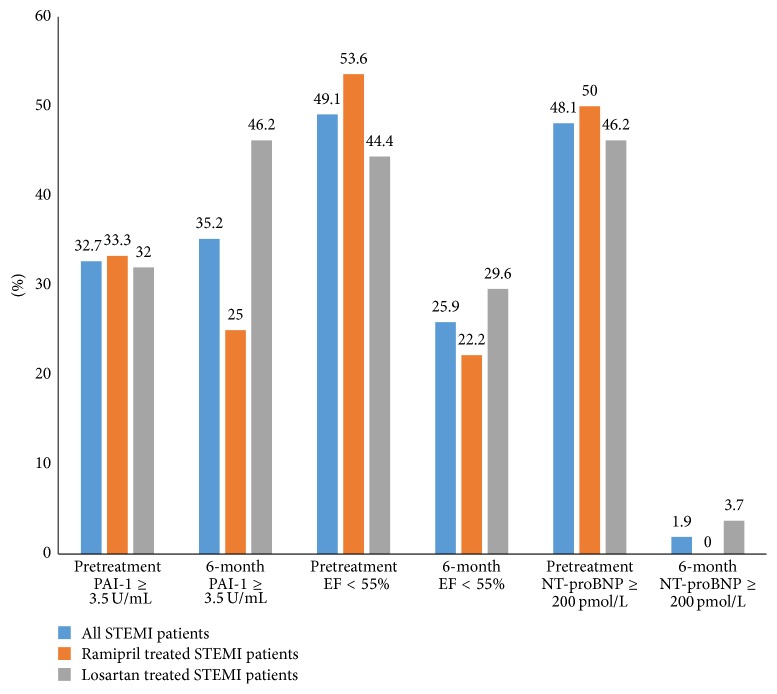
Proportions of all STEMI patients and ramipril and losartan treated STEMI patients with increased NT-proBNP and PAI-1 and decreased EF levels at baseline and six months later. NT-proBNP: N-terminal fragment of pro-brain-natriuretic peptide; PAI-1: plasminogen-activator-inhibitor type 1; EF: ejection fraction; SD: standard deviation, months in comparison to baseline, but PAI-1 and EF levels nonsignificantly.

Proportions of patients with NT-proBNP levels ≥ 200 pmol/L, EF < 55%, and PAI-1 ≥ 3.5 U/mL at baseline and after six months are displayed in Figure 2. Between STEMI patients treated with ramipril and losartan, there were nonsignificant differences regarding increased NT-proBNP, PAI-1 levels, and decreased EF levels as illustrated in Figure 2.

Mean CT levels for CEPI are displayed in Table 3. In STEMI patients receiving either ramipril or losartan in addition to DAPT mean CT levels for CEPI after 8 weeks and 6 months were significantly increased in comparison to the control group, but between the ramipril and losartan group there were nonsignificant differences in mean CT levels after 8 weeks and 6 months of therapy as shown in Table 3.

## 4. Discussion

We demonstrated that in our asymptomatic patients after their first STEMI, treated by PPCI treatment by ramipril or losartan, exerted an equal effect upon NT-proBNP, PAI-1, and EF after six months as shown in Table 2 and Figure 2.

In addition we observed that both groups—treated with either ramipril or losartan—increased antiplatelet activity, measured by CT significantly when compared to controls as shown in Table 3. The control group consisted of STEMI patients, treated by DAPT only.

Several studies have demonstrated that the magnitude of NT-proBNP, released by an increased left ventricular wall stress induced by ischemia, strongly correlates with the size of acute ischemic necrosis in STEMI patients and its extension within the next few months [2, 26–28]. Even more, a decreased EF correlated with an increase in NT-proBNP over 100 pmol/L at baseline and after 6 months [27].

In our study the baseline NT-proBNP was estimated just before randomization—that is, approximately 20–24 hours after the start of chest pain. This is in accordance with the findings of several studies that the optimum timing to estimate prognostic levels of NT-proBNP should be 24–36 hours after the event [28].

When we stratified baseline NT-proBNP levels we observed that baseline NT-proBNP levels of ≥200 pg/mL were present in 48% of all STEMI patients, including 50% ramipril and 46.2% losartan treated patients. After 6-month treatment NT-proBNP levels were below 200 pg/L in >90% of STEMI patients—equally in the ramipril or losartan group. This effect upon NT-proBNP was already observed after 8 weeks of treatment with either ramipril or losartan in our previous study [4]. In our present study the effect of ramipril and losartan was even more pronounced after 6-month treatment.

In spite that fact that our STEMI patients, who were treated by PPCI 24 hours earlier, were asymptomatic at the start of random assignment to ramipril or losartan, baseline NT-proBNP levels ≥200 pg/mL were present in approximately 50% of patients. Luchner et al. demonstrated that NT-proBNP levels were higher in outpatients after myocardial infarction than in healthy controls, even in the absence of heart failure or significant systolic dysfunction. The reason might be most probably significant cardiac remodeling due to persistent renin-angiotensin-aldosterone system activation [27]. In addition, Weber et al. found that highest values of NT-proBNP were observed 24–36 hours after the start of chest pain, but admission levels were within normal. NT-proBNP levels strongly correlated with troponin T levels either on admission or later, confirming the release of NT-proBNP from ischemic cardiomyocytes [28]. This confirms the observations that NT-proBNP is released from myocardium as a response to ventricular wall stress, but also from ischemic cardiomyocytes [27, 28].

In our asymptomatic STEMI patients baseline mean troponin I level was of 3.9 *μ*g/L and a mean peak level 45.3 *μ*g/L, suggesting a moderate ischemic necrosis. In fact mean EF level was 53.5 ± 9.1% and baseline EF levels <55% in 49.1% of included STEMI patients—equally in ramipril and losartan groups (53.6% versus 44.4%). Mild systolic dysfunction improved gradually, but not earlier than six months later, when EF was <55% only in approximately 25% of STEMI patients—again equally in ramipril and losartan treated.

Brown et al. demonstrated in insulin-resistant hypertensives a greater decrease in PAI-1 antigen for ACE inhibition than for ARB after 6-week therapy, but the effect of both drugs (ramipril and losartan) was similar within the first 3-4 weeks, suggesting that ARBs may exert only a transient effect upon PAI-1 [29]. Regarding PAI-1 levels we demonstrated nonsignificant differences between STEMI patients treated with ramipril and losartan at baseline and after six months. Neither ramipril nor losartan affected PAI-1 levels significantly after 6 months.

In STEMI patients, in particular after PPCI with the use of stents, in particular DES, DAPT should be given for one year in order to prevent in-stent thrombosis and reinfarctions [1, 2, 12]. Novel antiplatelet agents such as ticagrelor and prasugrel are recommended as the first-choice ADP inhibitors in addition to ASA, as they more successfully prevent recurrent thrombosis [1, 2, 12]. However, our results suggest that the blockade of renin-angiotensin-aldosterone system either by ramipril or losartan in addition to DAPT would improve antiplatelet activity further, as measured by CT after 8 weeks or 6 months in comparison to control groups. In contrast to our results, previous studies demonstrated that ARBs exert stronger antiplatelet effect than ACE inhibitors [7]. Schieffer et al. showed in a randomized trial in coronary patients after PCI that blockade of renin-angiotensin-aldosterone system with either ACE inhibitor or ARB reduced equally some of the inflammatory markers, but levels of IL-6, hsCRP, and platelet aggregation were reduced only by ARB, suggesting more pronounced antiplatelet effect by long-term use of an ARB than of an ACE inhibitor [7].

In our patients antiplatelet effect was estimated by CT and measured by PFA-100. This method is highly dependent on the von Willebrand factor (vWF) binding to the platelet membrane glycoprotein (GP) receptors Ib/IX/V and IIb/IIIa under high shear, but also on platelet count and hematocrit. Platelet count in all our STEMI patients and in the control group was normal [13, 15, 25]. Paniccia et al. compared several aggregometric methods in high-risk coronary patients, undergoing PCI, and discovered that PFA-100 CT, measured by CEPI cartridges, correlated significantly with other validated aggregometric methods [13].

Gianetti et al. in a randomized trial of standard versus tailored DAPT in STEMI patients measured platelet function also by PFA-100 with CEPI cartridges and concluded that this simple method could be a useful tool in acute coronary patients to identify high-on-treatment platelet reactivity [30].

PFA-100 is an example of a global platelet function assay that measures multiple platelet functions, including platelet adhesion and aggregation [14]. In spite of a need for better standardization, it can identify patients with high on-treatment platelet reactivity [15, 30]. Our STEMI patients were all treated by DAPT and then randomized to ramipril and losartan. It seems that adding ramipril or losartan significantly prolonged CT, which may reflect a significant decrease in platelet reactivity after blockade of renin-angiotensin-aldosterone system by ramipril or losartan.

Ono et al. demonstrated, in an experimental model, that losartan's antiplatelet effect is due to inhibition of platelet adhesion and aggregation via glycoprotein VI and was associated with losartan's molecular structure—phenyl group with the tetrazole ring [31]. In an animal study, Kalinowski et al. demonstrated that prevention of platelet adhesion and aggregation by losartan, its metabolite EXP3174, and valsartan are linked to NO release. At the same time, tested drugs could release NO directly, acting on either resting platelets or cultured endothelial cells [32].

On the other hand, Krämer et al. demonstrated, in hypertensive patients without coronary artery disease, that anti-inflammatory and antiplatelet properties of losartan were mainly mediated by another metabolite EXP3179 [9].

Therefore, in clinical settings, losartan may contribute to the prevention of coronary thrombosis and future coronary events by these two mechanisms, which are independent of its effect upon PAI-1. This effect would be of particular significance after STEMI in the long term, when DAPT is already discontinued.

Our results suggest that neurohormonal blockade by ramipril and losartan was equal after 6 months regarding decreased NT-proBNP levels. Systolic function—as measured by EF—in our asymptomatic patients was restored equally after six months by either ramipril or losartan. In addition antiplatelet activity was more significant when losartan or ramipril was added to DAPT in patients after STEMI, treated by PPCI, resulting in a similar and significant decrease in mean CT.

Our conclusions are that in asymptomatic STEMI patients after PPCI ramipril and losartan exert an important additional antiplatelet effect.

## Figures and Tables

**Table 1:** Baseline clinical and laboratory data of all STEMI patients and comparisons between patients treated with ramipril and losartan.

Baseline clinical and laboratory data	All patients (*n* = 55)	Ramipril (*n* = 28)	Losartan (*n* = 27)	*p*
Mean age ± SD (years)	58.7 ± 9.9	59.1 ± 11.2	58.3 ± 8.4	0.774
Mean BMI ± SD	27.4 ± 4.3	26.9 ± 3.4	27.8 ± 5.0	0.447
Mean peak TnI ± SD (*μ*g/L)	45.3 ± 38.5	42.2 ± 37.7	48.4 ± 39.9	0.553
Mean admission TnI ± SD (*µ*g/L)	4.0 ± 8.1	4.6 ± 8.5	3.4 ± 7.7	0.588
Time to PPCI ± SD (hours)	4.7 ± 3.8	5.4 ± 4.2	4.0 ± 3.1	0.156

STEMI: ST-elevation myocardial infarction; TnI: Troponin I; SD: standard deviation; PPCI: primary percutaneous coronary intervention.

**Table 2:** Clinical data of all STEMI patients at baseline and after 6 months and comparisons between STEMI patients treated with ramipril and losartan after six months of therapy.

Clinical data (mean ± SD)	All (*n* = 55)	Ramipril (*n* = 28)	Losartan (*n* = 27)	*p*
NT-proBNP (pg/mL)				
Before treatment	222.4 ± 189.1	211.7 ± 181.1	233.9 ± 200.2	0.671
6 months after treatment	40.3 ± 56.1^*∗*^	29.6 ± 21.4^*∗*^	51.0 ± 75.6^*∗*^	0.163
PAI-1 activity (U/mL)				
Before treatment	2.8 ± 2.1	2.6 ± 2.1	3.1 ± 2.1	0.408
6 months after treatment	2.8 ± 1.9	2.4 ± 1.8	3.1 ± 1.9	0.163
EF (%)				
Before treatment	53.5 ± 9.1	53.5 ± 9.4	53.6 ± 9.0	0.971
6 months after treatment	56.8 ± 8.3	57.0 ± 8.1	56.6 ± 8.5	0.858

Within group analysis: ^*∗*^ *p* < 0.05.

NT-proBNP: N-terminal fragment of pro-brain-natriuretic peptide; PAI-1: plasminogen activator inhibitor type 1; EF: ejection fraction.

**Table 3:** Closure times for collagen/epinephrine and platelet counts in all STEMI patients and comparisons between ramipril and losartan treated STEMI patients after 8 weeks and six months of therapy.

Mean ± SD	All (*n* = 55)	Ramipril (*n* = 28)	Losartan (*n* = 27)	Control (*n* = 9)
After 8-week treatment				
CT (sec)	239.8 ± 73.5^*∗*^	235.7 ± 84.6^*∗*^	244.7 ± 59.4^*∗*^	158.1 ± 80.3^*∗*^
Platelet count (1 × 10^9^/L)	202.5 ± 85.7^*∗*^	190.7 ± 47.5^*∗*^	216.7 ± 116.2	278.6 ± 158.1^*∗*^
After 6-month treatment				
CT (sec)	226.7 ± 80.3^*∗*^	226.9 ± 76.8^*∗*^	226.3 ± 85.0^*∗*^	158.1 ± 80.3^*∗*^
Platelet count (1 × 10^9^/L)	195 ± 70.9^*∗*^	190.9 ± 44.3^*∗*^	199.3 ± 91.6	278.6 ± 158.1^*∗*^

CT: closure time for collagen/epinephrine; ^*∗*^there is a statistically significant *p* value (<0.05) between the control group and other groups.
